# Predicting multiple linear stapler firings in double stapling technique with an MRI-based deep-learning model

**DOI:** 10.1038/s41598-023-46225-6

**Published:** 2023-11-02

**Authors:** Zhanwei Fu, Shuchun Li, Lu Zang, Feng Dong, Zhenghao Cai, Junjun Ma

**Affiliations:** Department of General Surgery, Ruijin Hospital, Shanghai Jiao Tong University School of Medicine, No. 197 Ruijin Er Road, Shanghai, 200025 People’s Republic of China

**Keywords:** Cancer, Gastroenterology, Medical research

## Abstract

Multiple linear stapler firings is a risk factor for anastomotic leakage (AL) in laparoscopic low anterior resection (LAR) using double stapling technique (DST) anastomosis. In this study, our objective was to establish the risk factors for ≥ 3 linear stapler firings, and to create and validate a predictive model for ≥ 3 linear stapler firings in laparoscopic LAR using DST anastomosis. We retrospectively enrolled 328 mid–low rectal cancer patients undergoing laparoscopic LAR using DST anastomosis. With a split ratio of 4:1, patients were randomly divided into 2 sets: the training set (n = 260) and the testing set (n = 68). A clinical predictive model of ≥ 3 linear stapler firings was constructed by binary logistic regression. Based on three-dimensional convolutional networks, we built an image model using only magnetic resonance (MR) images segmented by Mask region-based convolutional neural network, and an integrated model based on both MR images and clinical variables. Area under the curve (AUC), sensitivity, specificity, accuracy, positive predictive value (PPV), and Youden index were calculated for each model. And the three models were validated by an independent cohort of 128 patients. There were 17.7% (58/328) patients received ≥ 3 linear stapler firings. Tumor size ≥ 5 cm (odds ratio (OR) = 2.54, 95% confidence interval (CI) = 1.15–5.60, p = 0.021) and preoperative carcinoma embryonic antigen (CEA) level > 5 ng/mL [OR = 2.20, 95% CI = 1.20–4.04, p = 0.011] were independent risk factors associated with ≥ 3 linear stapler firings. The integrated model (AUC = 0.88, accuracy = 94.1%) performed better on predicting ≥ 3 linear stapler firings than the clinical model (AUC = 0.72, accuracy = 86.7%) and the image model (AUC = 0.81, accuracy = 91.2%). Similarly, in the validation set, the integrated model (AUC = 0.84, accuracy = 93.8%) performed better than the clinical model (AUC = 0.65, accuracy = 65.6%) and the image model (AUC = 0.75, accuracy = 92.1%). Our deep-learning model based on pelvic MR can help predict the high-risk population with ≥ 3 linear stapler firings in laparoscopic LAR using DST anastomosis. This model might assist in determining preoperatively the anastomotic technique for mid–low rectal cancer patients.

## Introduction

Colorectal cancer (CRC) is the third most prevalent cancer worldwide with high rate of cancer mortality, while approximately one third of all CRCs occur in rectum^1^. Total mesorectal excision (TME) is the primary treatment for localized rectal cancer patients, and has high rate of cancer control^2,3^. It is a widely applied surgical technique to perform laparoscopic low anterior resection (LAR) using double-stapling technique (DST) anastomosis^4,5^.

In mid–low rectal cancer patients, anastomotic leakage (AL) is the most common postoperative complication after LAR^6^. In addition to increasing the incidence of reoperation and mortality, AL may even negatively affect long-term survival^7–9^. Despite advances in surgical techniques and anastomotic equipment over the past few decades^10–12^, there has been no significant decrease in the incidence of AL after LAR^13^.

DST simplifies colorectal reconstruction in LAR, especially in laparoscopic surgery, but the incidence of postoperative AL was not reduced by the application of this technique^14,15^. Several studies have proved that ≥ 3 linear stapler firings in laparoscopic LAR is an independent risk factor for AL^16–18^. Thus, the Chinese expert consensus statement proposes ≤ 2 linear stapler firings in LAR surgery^19^. To avoid multiple linear stapler firings, a predictive model should be created to predict ≥ 3 linear stapler firings in anastomosis, and alternative techniques could be considered.

Several studies have shown that pelvic anatomical features (such as the anteroposterior diameter and the transverse diameter of the pelvic outlet, the anteroposterior diameter of the pelvic inlet) are related to surgical difficulty and the number of the linear stapler firings^20–22^. Those studies mainly measured pelvic parameters, but did not include the influence of rectal and mesenteric conditions. How to effectively integrate above parameters to predict ≥ 3 linear stapler firing needs further research.

With the advancement of imaging technology, pelvic magnetic resonance imaging (MRI) is a preferred tool for local staging of mid-low rectal cancer before surgery^23,24^. MRI can obtain relevant parameters of tumor, meso-rectum and pelvis comprehensively and accurately. Mask region-based convolutional neural network (Mask R-CNN)^25^ and three-dimensional convolutional network (C3D)^26^ image recognition are current techniques for advanced-recognition artificial intelligence (AI), and have been applied in various medical fields^27–29^. With advanced deep-learning technology, pelvic MRIs’ complex data can be identified, extracted, analyzed, and integrated efficiently, and we can create a predictive model to screen out high-risk patients with ≥ 3 linear stapler firings based on the database.

In this study, we aimed to establish the risk factors for ≥ 3 linear stapler firings in laparoscopic LAR using DST anastomosis, and to create and validate a predictive model for ≥ 3 linear stapler firings with deep-learning technology based on MRI.

## Methods

### Patients

A total of 328 mid–low rectal cancer patients who underwent laparoscopic LAR at Ruijin Hospital Affiliated to Shanghai Jiaotong University School of Medicine, China, between January 2016 and June 2021 were retrospectively analyzed as the deep-learning set. Clinical data were obtained from Ruijin hospital database and medical records. All methods were performed in accordance with the relevant guidelines and regulations, and the study was approved by the Medical Ethics Committee of Ruijin Hospital (No. 2019-82). The need for informed consent was waived by Ethics Committee of Ruijin Hospital. With a split ratio of 4:1, 260 patients were divided into the training set and 68 patients were divided into the testing set on the basis of an unbiased random sampling method. The prospective validation set comprised 128 patients from an independent clinical trial in our institution (Transanal versus laparoscopic total mesorectal excision for rectal cancer, ClinicalTrials.gov Identifier: NCT03359616).

The inclusion criteria were: (1) histopathologically confirmed rectal cancer; (2) pelvic MRI examination within 14 days before surgery; (3) tumor distance from the anal verge ≤ 10 cm; and (4) laparoscopic LAR with DST anastomosis. The exclusion criteria were: (1) LAR without anastomosis (e.g., Hartmann’s operation); (2) anastomotic techniques rather than DST (e.g., manual rectal anastomosis through the anus); (3) number of linear stapler firings was not recorded on surgical reports; and (4) robotic surgery.

### Surgical procedure

Laparoscopic LAR was performed by the gastrointestinal surgery team with experience in completing more than 100 rectal cancer operations every year. The laparoscopic LAR surgical procedure was carried out in strict accordance with the national guidelines for laparoscopic radical resection of CRC (2018 edition). During dissection of the distal rectum, the surgeon manually fired endoscopic linear staplers (Endo-GIA™ Ultra Universal Stapler Reload with Tri-stapler™ Technology; Covidien LLC), which was loaded with 60- or 45-mm staple cartridges which have three types of heights: 3.0, 3.5 and 4.0 mm.

### Data collection and model building

We collected clinical data that potentially correlated with the number of linear staplers used in surgery, including baseline characteristics, including age, gender and body mass index (BMI); biochemical data, including hemoglobin, albumin, and carcinoma embryonic antigen (CEA); and tumor characteristics, including tumor distance from the anal verge, tumor stage, tumor size and circumferential resection margin (CRM). A predictive model of ≥ 3 linear stapler firings was constructed by binary logistic regression. The variables of the clinical model included: gender, BMI, serum CEA level (> or ≤ 5 ng/mL), tumor distance from the anal verge, tumor size and CRM.

### MRI and target area labeling

During MRI, patients were in the supine position and scanned using a Philips INGENIA™ scanner with 3.0 T field strength. The pelvic phased-array surface coil covered from the aortic bifurcation to the anal verge. The scanning parameters were: layer thickness 5 mm; field of view 250 × 340 × 166 mm; echo time 80 ms; repetition time 3565 ms; and image matrix 312 × 357.

**Figure 1:**
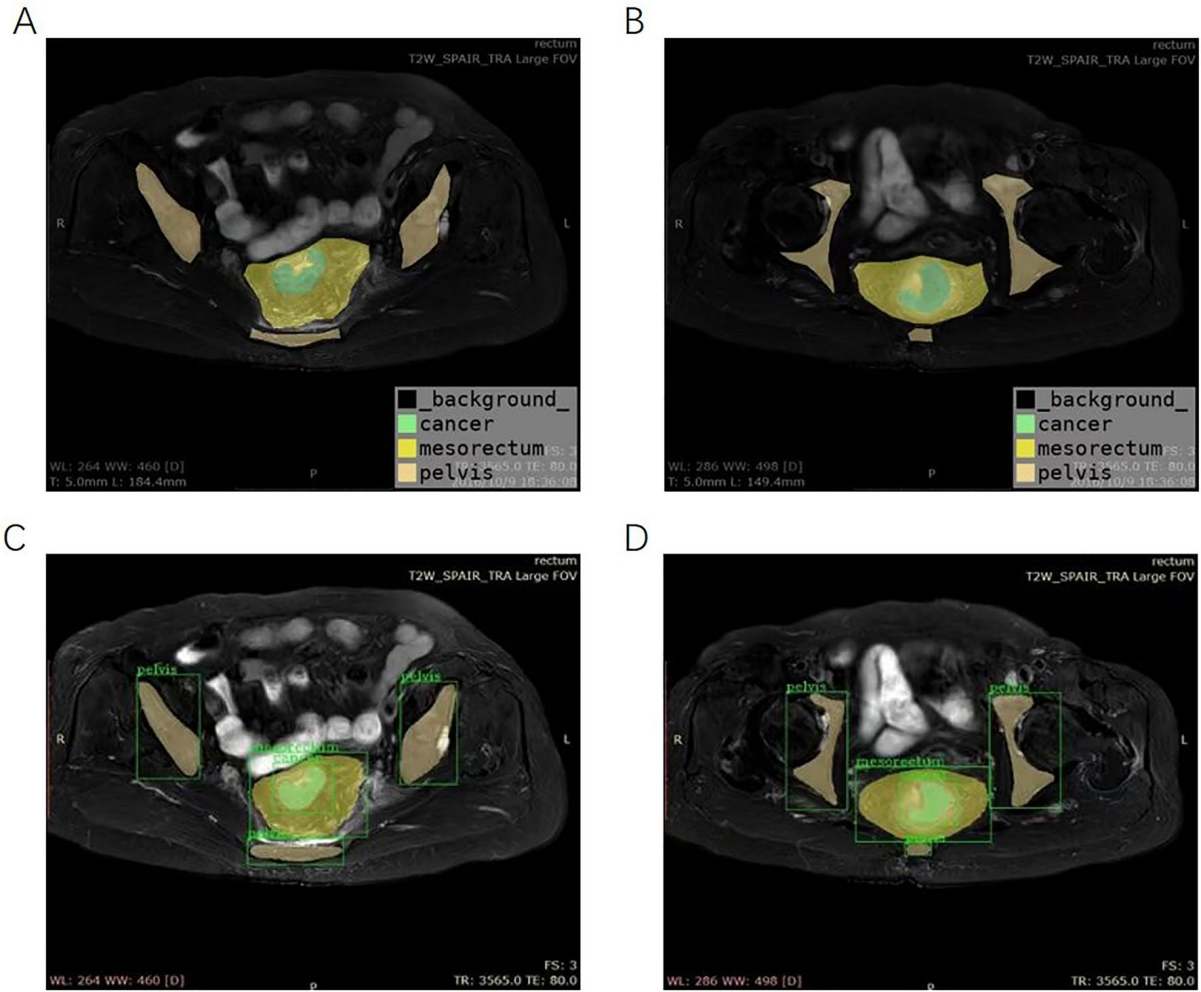
Examples of target regions. (**A**,**B**) manually labeled; (**C**,**D**) segmented by Mask region-based convolutional neural network (Mask R-CNN) based model (the regions of tumor body, mesorectum, and pelvis were represented by green, yellow, and drab, respectively).

With the Picture Archiving and Communication System, fat-suppressed fast spin-echo (FSE) T2-weighted sequences in the axial plane of the pelvis were used for image segmentation. Then, pelvic MRI specialists who have > 10 years of experience built an image database by an online annotation tool called Labelme (labelme.csail.mit.edu/)^30^, and labeled three kinds of target area on each of the T2-weighted images (tumor body, mesorectum, and pelvis represented by green, yellow, and drab, respectively, Fig. 1A,B). Then, all data were transformed into a COCO dataset for segmentation experiments^31^.

### Segmentation model based on Mask R-CNN

As an effective two-stage detection and segmentation algorithm, Mask R-CNN was adopted to identify and segment the three kinds of target areas in the image (Fig. 1C,D). In the first stage, the ResNet-101-FPN network served as the backbone to extract multiscale and discriminative feature maps. The Region Proposal Network scanned the feature map in a sliding-window and selected the rough detection rectangle that contained the object. After the regions of interest alignment process, the candidate regions entered the next stage. This consisted of three functional branches: classification, detection and segmentation, based on fully connected layers and convolutional layers. We trained the Mask R-CNN network on the training set for 200 epochs, and evaluated the performance of the testing set with standard COCO metrics. We evaluated the trained Mask R-CNN model, obtained average precision (AP) by calculating the precision–recall curve under different intersection-over-union thresholds, and then calculated the three types of targets. The respective AP values of the regions were weighted to obtain the class-wide mean average precision (mAP). mAP > 50 indicated that the model performed well^32^.

### Deep-learning model based on C3D

We used C3D networks to address 24 images of a case simultaneously and learn 3D spatial features. The C3D network consisted of eight 3D-convolution layers, a softmax layer, two fully connected layers and five pooling layers. It took the entire image of the case as input and output the probability of ≥ 3 linear stapler firings, and the sample with probability > 50% (empirical value) was judged as positive. The C3D network was trained until convergence (~ 1000 epochs) and evaluated the performance of the deep-learning model. In the training set, two C3D-based models were trained, including an image model using only MR images and an integrated model based on both MR images and the six clinical variables in clinical model. In the testing set, the clinical model, image model and integrated model were examined, and receiver operating characteristic (ROC) curves were plotted. Area under the curve (AUC), sensitivity, specificity, accuracy, positive predictive value (PPV), and Youden index were calculated.

### Prospective validation

**Figure 2:**
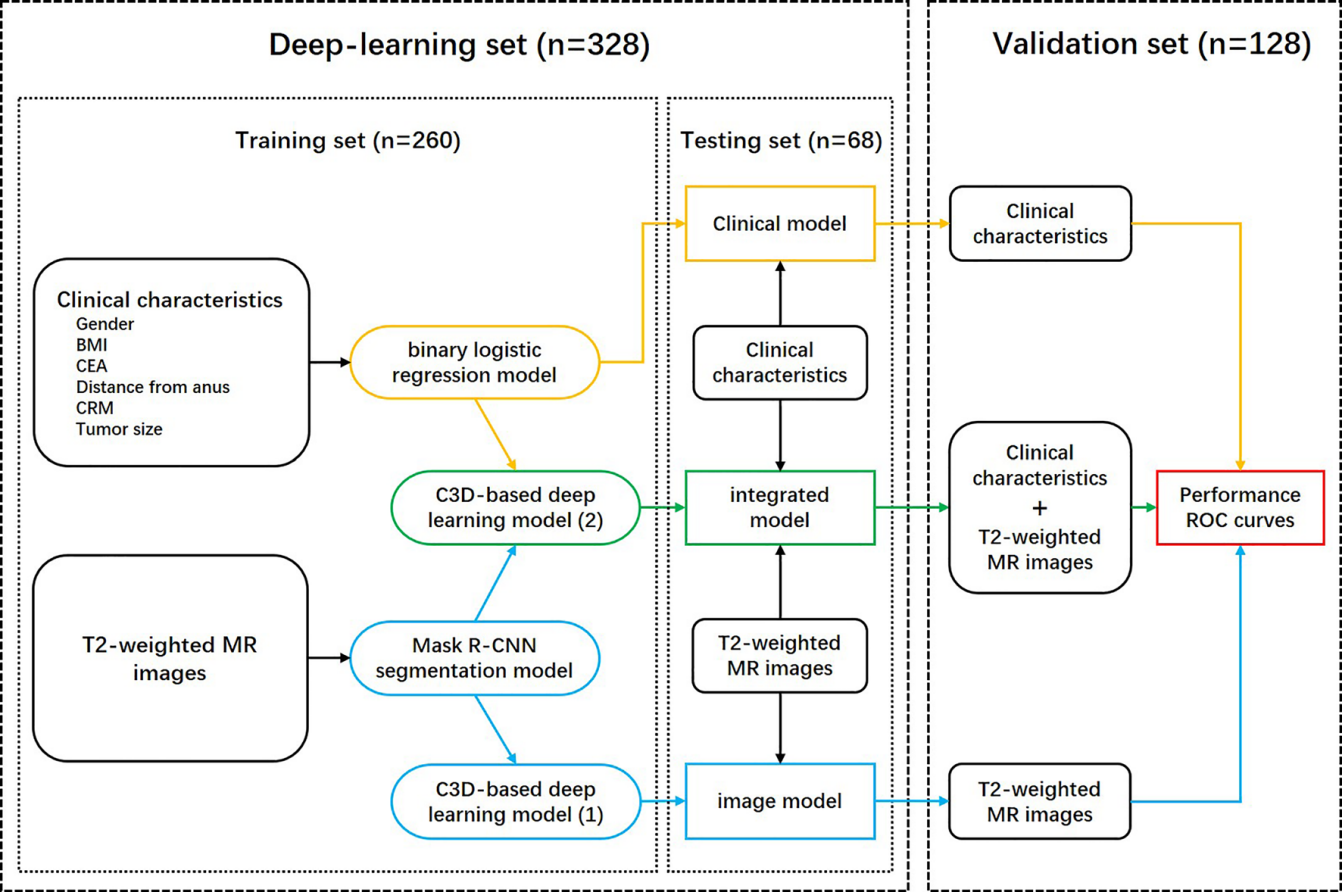
Flow chart of the design. BMI: Body mass index; CEA: Carcinoembryonic antigen; CRM: Circumferential resection margin; MR: Magnetic resonance; Mask R-CNN: Mask region-based convolutional neural network.

We used clinical data and T2-weighted images of patients from the validation set to validate the predictive performance of the above three models. ROC curves were plotted, and sensitivity, specificity, accuracy, Youden index, PPV, and AUC were calculated. The flow chart of the design is shown in Fig. 2.

### Statistical analysis

Statistical analysis was performed using SPSS (version 25.0). Categorical variables were analyzed by Fisher’s exact test or Pearson’s chi-square. In our binary logistic regression models, only factors with a P value < 0.10 in the univariate analysis were entered into the multivariate analysis. All tests were two-sided, and differences were considered statistically significant at p < 0.05.

## Results

### Clinical characteristics of patients in deep-learning set

The 328 patients had a median age of 63 (24–87) years, including 227 men and 101 women. The proportion of patients who received ≥ 3 linear stapler firings was 17.7% (58/328). Clinical characteristics of patients with ≥ 3 firings of the linear stapler and those with ≤ 2 firings in the deep-learning set were compared (Table 1). There were no significant differences in age, gender, BMI, diabetes mellitus, neoadjuvant chemoradiotherapy ratio, operation time, hemoglobin, albumin, tumor distance from the anal verge, T stage and N stage between the two groups. Patients with ≥ 3 firings showed significantly higher incidence of AL than those with ≤ 2 firings (p = 0.021), and patients with ≥ 3 firings of the linear stapler showed significantly higher CEA level (p = 0.007), larger tumor size (p = 0.004) and higher rate of positive CRM (p = 0.014). In univariate and multivariate analyses, tumor size ≥ 5 cm (odds ratio (OR) = 2.54, 95% confidence interval (CI) = 1.15–5.60, p = 0.021) and serum CEA > 5 ng/mL [OR = 2.20, 95% CI = 1.20–4.04, p = 0.011] were independent risk factors associated with ≥ 3 linear stapler firings (Table 2).

**Table 1:** Clinical characteristics of patients in deep-learning set.

Number of linear stapler firings	≥ 3	≤ 2	p value
	n = 58 (17.7%)	n = 270 (82.3%)	
Baseline characteristics			
Age [y]			0.218
> 70	16 (27.6)	54 (20.0)	
≤ 70	42 (72.4)	216 (80.0)	
Gender, n (%)			0.084
Male	46 (79.3)	181 (67.0)	
Female	12 (20.7)	89 (33.0)	
BMI [kg/m^2^]			0.745
> 25	17 (29.3)	72 (26.7)	
≤ 25	41 (70.7)	198 (73.3)	
Diabetes mellitus, n (%)			0.873
Yes	9 (15.5)	32 (11.9)	
No	49 (84.5)	238 (88.1)	
nCRT, n (%)			0.709
Yes	14 (24.1)	71 (26.3)	
No	44 (75.9)	199 (73.7)	
Operation time [min]			0.246
> 150	32 (55.2)	124 (45.9)	
≤ 150	26 (44.8)	146 (54.1)	
Anastomotic leakage, n (%)			0.021
Yes	14 (24.1)	34 (12.6)	
No	44 (75.9)	236 (87.4)	
Biochemical data			
Hemoglobin [g/L]			0.893
< 120	12 (20.7)	58 (21.5)	
≥ 120	46 (79.3)	212 (78.5)	
Albumin [g/L]			0.207
< 35	8 (13.8)	22 (8.1)	
≥ 35	50 (86.2)	248 (91.9)	
CEA [ng/mL]			0.007
> 5	26 (44.8)	71 (26.3)	
≤ 5	32 (55.2)	199 (73.7)	
Tumor characteristics			
Distance from anus [cm]			0.138
< 5	4 (6.9)	39 (14.4)	
≥ 5	54 (93.1)	231 (85.6)	
Tumor size [cm]			0.004
≥ 5	14 (24.1)	26 (9.6)	
< 5	44 (75.8)	244 (90.4)	
T stage, n (%)			0.865
T ≤ 2	14 (24.1)	68 (25.2)	
T3-4	44 (75.9)	229 (84.8)	
N stage, n (%)			0.663
N0	30(51.7)	152 (56.3)	
N+	28(48.3)	118 (43.7)	
CRM (evaluated by MRI), n (%)			0.014
Positive	20 (34.5)	52 (19.3)	
Negative	38 (65.5)	218 (80.7)	

MRI: magnetic resonance imaging; CRM: circumferential resection margin; N: node; T: tumor; CEA: carcinoma embryonic antigen; nCRT: neoadjuvant chemoradiotherapy; BMI: body mass index.

**Table 2:** Risk factors of ≥ 3 linear stapler firings in deep-learning set.

Factors	Univariate analysis		Multivariate analysis	
	OR (95% CI)	p value	OR (95% CI)	p value
Age [y] (≥ 70/ < 70)	1.45 (0.82, 2.56)	0.202	N.A	N.A
Gender (male/female)	1.89 (0.95, 3.74)	0.069	1.82 (0.90, 3.71)	0.098
BMI [kg/m^2^] (≥ 25/ < 25)	1.14 (0.61, 2.13)	0.681	N.A	N.A
Diabetes mellitus (Y/N)	1.37 (0.61, 3.04)	0.445	N.A	N.A
nCRT (Y/N)	0.89 (0.46, 1.73)	0.734	N.A	N.A
Albumin [g/L] (< 35/ ≥ 35)	1.80 (0.76, 4.28)	0.181	N.A	N.A
CEA [ng/mL] (> 5/ ≤ 5)	2.39 (1.33, 4.32)	0.004	2.20 (1.20, 4.04)	0.011
Distance from anus [cm] (< 5/ ≥ 5)	2.28 (0.78, 6.65)	0.132	N.A	N.A
Tumor size [cm] (≥ 5/ < 5)	2.99 (1.45, 6.16)	0.003	2.54 (1.15, 5.61)	0.021
CRM (evaluated by MRI) (+/−)	2.21 (1.19, 4.10)	0.012	1.66 (0.84, 3.25)	0.143

MRI: magnetic resonance imaging; CRM: circumferential resection margin; CEA: carcinoma embryonic antigen; nCRT: neoadjuvant chemoradiotherapy; BMI: body mass index; N.A.: not applicable; OR: odds ratio; CI: confidence interval.

### Predicting performance in testing set

**Figure 3:**
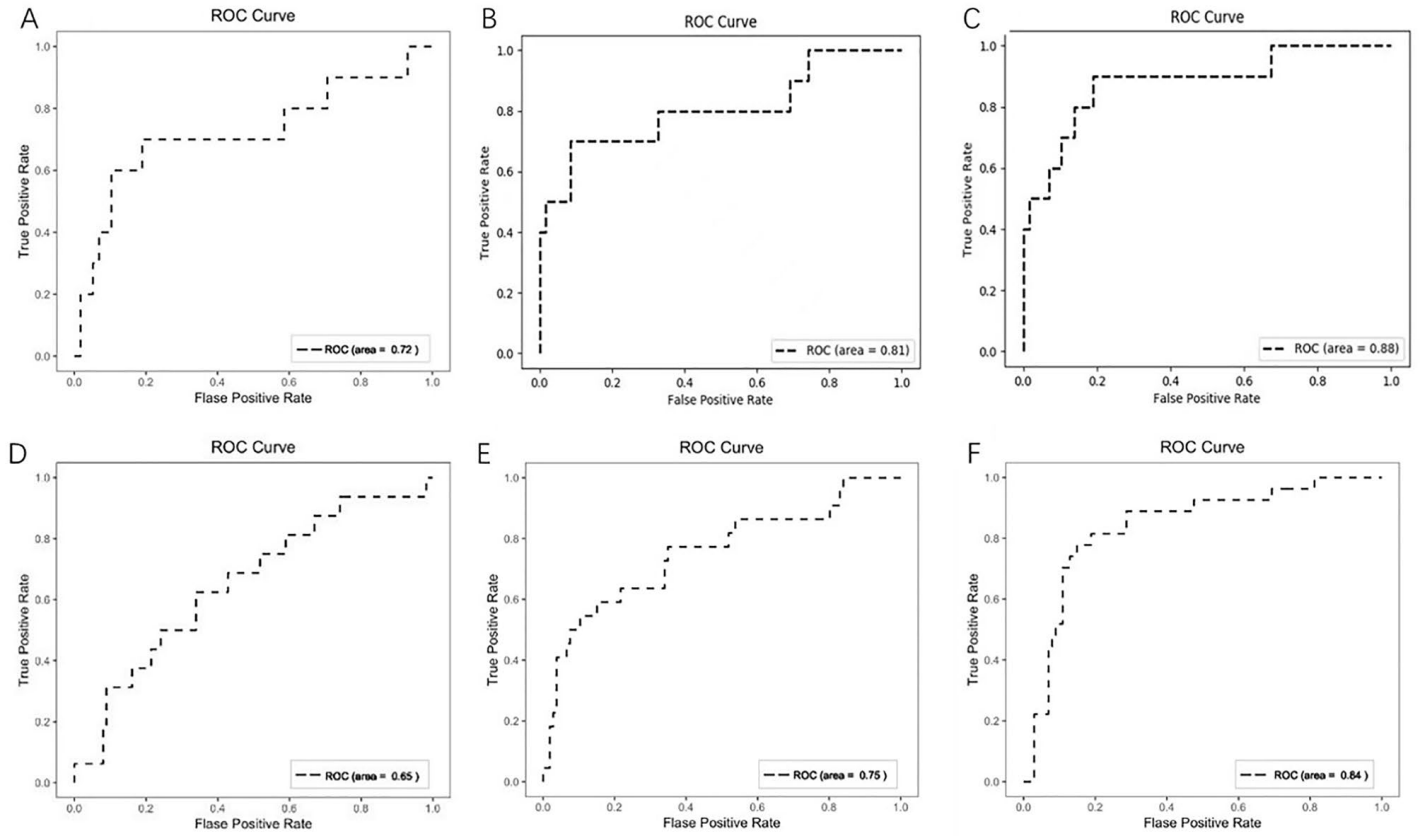
Receiver operating characteristic curves of the predictive models. (**A**) clinical model in deep-learning set; (**B**) image model in deep-learning set; (**C**) integrated model in deep-learning set; (**D**) clinical model in validation set; (**E**) image model in validation set; (**F**) integrated model in validation set.

The AUCs of the clinical, imaging and integrated models were obtained as 0.72, 0.81 and 0.88, respectively (Fig. 3A–C). The sensitivity, specificity, accuracy, PPV and Youden index of the clinical model were: 70.0%, 81.0%, 79.4%, 38.9%, and 0.51, respectively. The relevant indicators of the image model were: 50.0%, 98.3%, 91.2%, 83.3%, and 0.48, respectively. The relevant indicators of the integrated model were: 70.0%, 98.3%, 94.1%, 87.5%, and 0.68, respectively.

### Prospective validation

In the validation set, the proportion of patients who received ≥ 3 linear stapler firings was 12.5% (16/128). All related clinical characteristics between the deep-learning and validation sets were comparable (Table 3). The AUCs of the clinical, imaging and integrated models were obtained as 0.65, 0.75 and 0.84, respectively (Fig. 3D–F).

**Table 3:** Comparison of clinical characteristics between deep-learning set and validation set.

	Deep-learning set	Validation set	p value
	n = 328	n = 128	
Baseline characteristics			
Age [y]			0.139
> 70	70 (21.3)	36 (28.1)	
≤ 70	258 (78.0)	92 (71.9)	
Gender, n (%)			0.561
Male	227 (69.2)	83 (64.8)	
Female	101 (30.8)	45 (35.2)	
BMI [kg/m^2^]			0.250
> 25	89 (27.1)	42 (32.8)	
≤ 25	239 (72.9)	86 (67.2)	
Diabetes mellitus, n (%)			0.342
Yes	41 (12.5)	23 (18.0)	
No	287 (87.5)	105 (82.0)	
nCRT, n (%)			0.201
Yes	85 (25.9)	41 (32.0)	
No	243 (74.1)	87 (68.0)	
Number of linear stapler cartridges, n (%)			0.204
≥ 3	58 (17.7)	16 (12.5)	
≤ 2	270 (82.3)	112 (87.5)	
Anastomotic leakage, n (%)			0.090
Yes	48 (14.6)	11 (8.6)	
No	280 (85.4)	117 (91.4)	
Biochemical data			
Albumin [g/L]			0.356
< 35	97 (29.6)	32 (25.0)	
≥ 35	231 (70.4)	96 (75.0)	
CEA [ng/mL], n (%)	(Missing = 5)		0.301
> 5	97 (29.6)	32 (25.0)	
≤ 5	226 (68.9)	96 (75.0)	
Tumor characteristics			
Distance from anus [cm]			0.637
< 5	43 (13.1)	14 (10.9)	
≥ 5	285 (86.9)	114 (89.1)	
Tumor size [cm], n (%)			0.130
≥ 5	40 (12.2)	9 (7.0)	
< 5	288 (87.8)	119 (93.0)	
CRM (evaluated by MRI), n (%)			0.092
Positive	72 (22.0)	19 (14.8)	
Negative	256 (78.0)	109 (69.5)	

MRI: magnetic resonance imaging; CRM: circumferential resection margin; CEA: carcinoma embryonic antigen; nCRT: neoadjuvant chemoradiotherapy; BMI: body mass index.

The sensitivity, specificity, accuracy, PPV and Youden index of the clinical model were: 62.5%, 66.1%, 65.6%, 21.0%, and 0.29, respectively. The relevant indicators of the image model were: 68.8%, 95.5%, 92.1%, 68.8%, and 0.64, respectively. The relevant indicators of the integrated model were: 68.8%, 97.3%, 93.8%, 78.5%, and 0.66, respectively.

## Discussion

In this study, we built an MRI-based deep-learning model to predict ≥ 3 linear stapler firings in LAR using DST anastomosis. This model aimed to help determine the surgical strategy for mid–low rectal cancer patients by predicting the probability of ≥ 3 firings of the linear stapler before surgery. Thus, we can reduce the occurrence of AL by using other more suitable anastomosis techniques. Our findings suggest that clinical information alone may not be sufficient to predict cases with ≥ 3 firings of the linear stapler. Compared with the clinical or image model, the integrated model that combined clinical information with pelvic MR images achieved better AUC and higher PPV.

LAR using DST anastomosis is currently a widely applied surgical technique for mid–low rectal cancer, a series of high-quality randomized controlled trials has confirmed its feasibility and safety^5,33^. The technique greatly reduces the difficulty of reconstruction of the digestive tract. However, some studies have reported that multiple linear stapler firings is closely related to AL^17,34^, and AL is more likely to occur at the intersection of two staples^35^. In some cases, due to the limitation of the pelvic space or thickness of the rectum, the surgeons have to trigger more linear stapler firings during rectal dissection^36^.

In the high-risk populations with ≥ 3 linear stapler firings, other anastomosis techniques rather than DST anastomosis could be considered, such as transanal anastomosis after transanal transection of the rectum^37^ and manual purse-string suture after endoluminal transection of the rectum (e.g., Transanal total mesorectal excision)^38^. Although some studies have shown that the above techniques do not reduce the incidence of AL^39^, these techniques can minimize the anastomotic difficulty in patients with a narrow pelvis and avoid excessive use of linear stapler firings.

Akiyoshi et al. used clinical data and pelvic parameters to predict surgical difficulty and the incidence of AL in patients undergoing LAR using DST anastomosis. They found that tumor distance from the anal verge, BMI, pelvic outlet, and tumor invasive depth were independent predictors of operation time and occurrence of AL^10^. Foo et al. reported a predictive model for predicting ≥ 3 linear stapler firings. The model included the following parameters: tumor distance from the anal verge, gender, pelvic entrance, internodal distance and interspinous distance^40^. Compared with the above two studies, our predictive model has several advantages. (1) Using AI-based image segmentation, pelvic measurements can be identified comprehensively, rather than obtaining certain pelvic parameters separately. Thus, all anatomical features of the pelvis can be entirely integrated into the image model. (2) Clinically, the space between the pelvis, mesorectum and tumor mass affects the number of linear stapler firings. Our model takes into account not only pelvic parameters, but also the influence of meso-rectal factors and tumor conditions. (3) The predicting time of this AI-based warning model is only 100 ms. It greatly reduces the time and labor of manual measurement.

It should be noted that our study has some limitations. First, the cohort of 328 patients was too small for training the deep-learning model, further study with larger sample size is needed. Second, other technical factors that were difficult to quantify can also affect the number of linear stapler firings, such as the correct angle between the stapler and rectum and precompression before stapler firings^35,36^. Therefore, no 100% accuracy were achieved in our three models. Third, the number of linear stapler firings was just one of anastomotic factors related to AL, the circular end-to-end anastomosis, intersections of staple lines^41^, and the distance between the linear staple line^36^ were also risk factors for AL. Finally, our deep-learning traning is only performed on T2-weighted MR sequences. Other MR sequences or contrast-enhanced MRI could be investigated in future studies.

In conclusion, the pelvic MR-based deep-learning model can help identify the high-risk population with ≥ 3 linear stapler firings in laparoscopic LAR surgery. It might help determine the anastomotic technique for mid–low rectal cancer patients preoperatively. However, it is still necessary to verify its value through clinical application.

## Data Availability

The datasets used during the current study available from the corresponding author on reasonable request.
